# Bleeding and Blood Disorders in Clients of Voluntary Medical Male Circumcision for HIV Prevention — Eastern and Southern Africa, 2015–2016

**DOI:** 10.15585/mmwr.mm6711a6

**Published:** 2018-03-23

**Authors:** Lawrence E. Hinkle, Carlos Toledo, Jonathan M. Grund, Vanessa R. Byams, Naomi Bock, Renee Ridzon, Caroline Cooney, Emmanuel Njeuhmeli, Anne G. Thomas, Jacob Odhiambo, Elijah Odoyo-June, Norah Talam, Faustin Matchere, Wezi Msungama, Rose Nyirenda, James Odek, Jotamo Come, Marcos Canda, Stanley Wei, Alfred Bere, Collen Bonnecwe, Isaac Ang’Ang’A Choge, Enilda Martin, Dayanund Loykissoonlal, Gissenge J.I. Lija, Erick Mlanga, Daimon Simbeye, Stella Alamo, Geoffrey Kabuye, Joseph Lubwama, Nafuna Wamai, Omega Chituwo, George Sinyangwe, James Exnobert Zulu, Charles A. Ajayi, Shirish Balachandra, John Mandisarisa, Sinokuthemba Xaba, Stephanie M. Davis

**Affiliations:** ^1^Public Health Institute/CDC Global Health Fellow; ^2^Division of Global HIV and TB, CDC; ^3^Division of Blood Disorders, CDC; ^4^Independent Consultant, Boston, Massachusetts; ^5^Office of the U.S. Global AIDS Coordinator, Washington, D.C.; ^6^U.S. Agency for International Development, Washington, D.C.; ^7^Naval Health Research Center, Department of Defense, San Diego, California; ^8^National AIDS and STI Control Programme, Ministry of Health, Nairobi, Kenya; ^9^Division of Global HIV and TB (Kenya), CDC; ^10^U.S. Department of Defense, Kericho, Kenya; ^11^U.S. Department of Defense, Lilongwe, Malawi; ^12^Division of Global HIV and TB (Malawi), CDC; ^13^Ministry of Health, Department of HIV & AIDS, Lilongwe, Malawi; ^14^U.S. Agency for International Development, Lilongwe, Malawi; ^15^Ministério da Saúde, Programa Nacional de Controlo De ITS, HIV/SIDA, Maputo, Mozambique; ^16^Division of Global HIV and TB (Mozambique), CDC; ^17^Division of Global HIV and TB (South Africa), CDC; ^18^Department of Health, Pretoria, South Africa; ^19^U.S. Agency for International Development, Pretoria, South Africa; ^20^Ministry of Health, Community Development, Gender, Elderly and Children, Dar es Salaam, Tanzania; ^21^U.S. Agency for International Development, Dar es Salaam, Tanzania; ^22^Division of Global HIV and TB (Tanzania), CDC; ^23^Division of Global HIV and TB (Uganda), CDC; ^24^Division of Global HIV and TB (Zambia), CDC; ^25^U.S. Agency for International Development, Lusaka, Zambia; ^26^Ministry of Community Development, Mother and Child Health, Lusaka, Zambia; ^27^U.S. Agency for International Development, Harare, Zimbabwe; ^28^Division of Global HIV and TB (Zimbabwe), CDC; ^29^Ministry of Health and Child Care, Harare, Zimbabwe.

Male circumcision reduces the risk for female-to-male human immunodeficiency virus (HIV) transmission by approximately 60% (*1*) and has become a key component of global HIV prevention programs in countries in Eastern and Southern Africa where HIV prevalence is high and circumcision coverage is low. Through September 2017, the President’s Emergency Plan for AIDS Relief (PEPFAR) had supported 15.2 million voluntary medical male circumcisions (VMMCs) in 14 priority countries in Eastern and Southern Africa (*2*). Like any surgical intervention, VMMC carries a risk for complications or adverse events. Adverse events during circumcision of males aged ≥10 years occur in 0.5% to 8% of procedures, though the majority of adverse events are mild (*3*,*4*). To monitor safety and service quality, PEPFAR tracks and reports qualifying notifiable adverse events. Data reported from eight country VMMC programs during 2015–2016 revealed that bleeding resulting in hospitalization for ≥3 days was the most commonly reported qualifying adverse event. In several cases, the bleeding adverse event revealed a previously undiagnosed or undisclosed bleeding disorder. Bleeding adverse events in men with potential bleeding disorders are serious and can be fatal. Strategies to improve precircumcision screening and performance of circumcisions on clients at risk in settings where blood products are available are recommended to reduce the occurrence of these adverse events or mitigate their effects (*5*).

To ensure safety and quality of VMMC services and to inform policy updates, PEPFAR tracks qualifying notifiable adverse events* and investigates individual cases. The notification process started in July 2014 and initially only tracked fatal adverse events. Before 2015, two bleeding-related fatalities were reported, one attributable to suspected factor VIII deficiency and one to a hemorrhage of unknown cause. These deaths served, in part, as the impetus for broadening the adverse event notification process to include some nonfatal adverse events, including bleeding resulting in hospitalization for ≥3 days, in January 2015. When a qualifying notifiable adverse event occurs, the in-country office is notified and conducts an investigation including a review of the patient’s medical chart for relevant clinical data. Ultimately, these investigations are reviewed by the Office of the Global AIDS Coordinator, U.S. Department of State, which tracks adverse events and the outcomes of investigations. This report summarizes bleeding adverse events associated with PEPFAR-supported VMMC programs in the eight countries (Kenya, Malawi, Mozambique, South Africa, Tanzania, Uganda, Zambia, and Zimbabwe) that reported bleeding adverse events through the adverse event notification process during 2015–2016.

An estimated 4.58 million PEPFAR-supported VMMCs were performed in these eight countries during 2015–2016. A review of all VMMC-associated notifiable adverse events during this period identified 109 events, including 19 (17.4%) bleeding adverse events. These events accounted for the largest number of any reported PEPFAR VMMC program–associated adverse event resulting in ≥3 days of hospitalization.

In all 19 cases, the client received a conventional surgical circumcision. Among the clients who experienced a bleeding adverse event, the median age was 16 years (range = 10–59 years). One client had bleeding intraoperatively and was hospitalized immediately, 14 experienced bleeding within 3 days of circumcision (including two who had also had transient intraoperative bleeding), and four experienced bleeding at 7–10 days after the procedure. In two of the 19 cases, the client sought care at a clinic at least twice for bleeding and was sent home after basic interventions, before being hospitalized during a subsequent clinic visit. Among the 19 clients who experienced bleeding adverse events, eight received fresh frozen plasma, whole blood, or platelet transfusions. Because of limited availability, one client received a product type not originally chosen by the treating physician, and another had to wait a day for transfusion. Among the 13 cases for which the hospital stay was completed and documented, the average length of hospitalization was 14.7 days. Five clients experienced secondary infection requiring debridement, including one case of Fournier gangrene; all survived.

Among the 19 clients with a bleeding adverse event, seven received a diagnosis of a bleeding disorder or other hematologic abnormality, seven had an unconfirmed suspected bleeding disorder, and for five, no evidence was available that a bleeding disorder was considered (Table). Because availability of testing to confirm a bleeding disorder was limited, not all clients had a complete laboratory evaluation, and a diagnosis of a bleeding disorder was frequently based on clinical data only.

**TABLE:** Bleeding disorders and diagnoses among men undergoing voluntary medical male circumcision with notifiable bleeding adverse events — eight Eastern and Southern African countries,* 2015–2016

Bleeding disorder (no. of clients)	Diagnosis	Supporting test results or interventions	No. of clients
**Confirmed bleeding disorder or other hematologic abnormality (n = 7)**	Hemophilia	Undisclosed prior diagnosis of hemophilia	1
	Hemophilia	Clinical response to Factor VIII administration	1
	Hemophilia	Severe Factor VIII deficiency	1
	Thrombocytopenia	Etiology unclear	1
	No clinical diagnoses	Prolonged clotting and bleeding times	1
	Unspecified bleeding dyscrasia^†^	Abnormal clotting profile	1
	Chronic myeloid leukemia	Diagnosis made based on results of complete blood count, client referred for Philadelphia Chromosome analysis	1
**Unconfirmed suspected bleeding disorder (n = 7)**	None	None	4
	None	Complete blood count (results normal)	1
	None	Bleeding time, clotting time (results not documented)	1
	None	Blood sample sent to hematologist (results unknown)	1
	None	Received or were considered for outpatient hematology referral	3
**No evidence that bleeding disorder/abnormality considered (n = 5)**	None	Postoperative bleeding resulted in hospitalization for >3 days, but no documentation that a bleeding diagnosis was considered	5

* Kenya, Malawi, Mozambique, South Africa, Tanzania, Uganda, Zambia, and Zimbabwe.

^†^ Referred to hematologist for follow-up.

## Discussion

Circumcision might be a male’s first engagement with a medical procedure that challenges the coagulation cascade and potentially unmasks a bleeding disorder. Before undergoing VMMC, all clients are interviewed to ascertain a history of family or personal bleeding history (*6*), although the extent of screening varies. Seven of the 19 clients (or their guardians) did disclose a bleeding history but only after the adverse event had occurred. These clients might have concealed their history to receive the procedure or have been unaware of the relevance of their histories.

The two most common inherited bleeding disorders are hemophilia and von Willebrand disease (*7*), and data on prevalence of these bleeding disorders are limited in sub-Saharan Africa. In the 2015 World Federation of Hemophilia Annual Global Survey (*8*), the eight VMMC countries reporting hemophilia data (Ethiopia, Kenya, Lesotho, South Africa, Tanzania, Uganda, Zambia, and Zimbabwe) reported a combined prevalence of approximately 1.05 cases per 100,000 population, 4.5-fold lower than the reported prevalence of 5.79 cases per 100,000 in the United States. This suggests possible underreporting, because U.S. data do not identify differences in incidence based on race or ethnicity. Among these eight countries, Ethiopia and Lesotho have not reported a bleeding adverse event; Malawi and Mozambique have reported bleeding adverse events but have no reported bleeding disorder data. Similarly, the four VMMC countries that report data on von Willebrand disease (Ethiopia, Kenya, South Africa, and Uganda) report a combined prevalence of 0.29 cases per 100,000 population, approximately 14-fold lower than the reported prevalence of 4.31 cases per 100,000 in the United States.

Recognizing that cases of postoperative bleeding in VMMC clients might be attributable to unreported or undiagnosed bleeding disorders has led CDC and PEPFAR to support development of tools to address this challenge, including a rapid verbal screening tool (*6*) designed for low-resource settings and an Adverse Event Action Guide addressing management of bleeding and indications for bleeding disorder workup (*5*). The screening tool is meant to screen for indications of any type of bleeding abnormality, including hemophilia and von Willebrand disease. When a potential client is known or suspected to have a bleeding disorder, he is counseled that male family members should also be considered to have a positive bleeding disorder screen when seeking VMMC. Likewise, if any member of a client’s family is known to have any bleeding abnormalities, he is considered to have a positive screen. Mild or moderate bleeding disorders are not contraindications to VMMC, but in cases where clients are considered or suspected to have a bleeding disorder, the Adverse Event Action Guide advises that the procedure be conducted in a setting where blood products are available. Furthermore, bleeding disorders must be considered in clients with prolonged or recurring postoperative bleeding, even without a prior bleeding history (*5*).

The findings in this report are subject to at least four limitations. First, cases are limited to clients with external bleeding, not those with isolated hematoma, which might underestimate the prevalence of bleeding. Second, some bleeding adverse events might have resulted from other causes, such as unligated bleeding vessels, rather than bleeding disorders. Third, the completeness of adverse event reporting, as well as clinical information, is not known. Finally, the VMMC clients do not represent a random sample of their countries’ male populations, as clients must pass a health screen before circumcision, and not all males might choose to have VMMC. Data to analyze associations with surgeon experience or circumcision technique used are not available.

In resource-limited settings, health facilities might lack the capability to diagnose a bleeding disorder and might lack the blood products necessary for treatment. Preoperative screening and early adverse event recognition and patient transfer are critical, and the Adverse Event Action Guide recommends that VMMC providers who suspect bleeding disorders in clients with bleeding adverse events refer them for evaluation. Bleeding disorders in VMMC clients are uncommon but important, and both careful preoperative screening and a high index of suspicion in bleeding adverse events are necessary to ensure client safety.

**Summary**

**What is already known about this topic?**

Voluntary medical male circumcision (VMMC) is a key component of human immunodeficiency virus (HIV) infection prevention programs in 14 Eastern and Southern African countries. The President’s Emergency Plan for AIDS Relief (PEPFAR) tracks notifiable adverse events in PEPFAR-supported programs to identify risks and improve practice.

**What is added by this report?**

A review of adverse event data reported to PEPFAR from VMMC programs in eight countries during 2015–2016 identified 19 cases of bleeding resulting in hospitalization of ≥3 days among 109 notifiable adverse events (17.4%); this was the most commonly reported adverse event. Among the 19 bleeding adverse events reported, seven occurred in clients who were later confirmed to have a bleeding disorder or nonspecific/other hematologic abnormality.

**What are the implications for public health practice?**

Efforts to improve precircumcision screening are intended to reduce the occurrence of bleeding adverse events by identifying clients who might have signs of a bleeding disorder. Clients considered or suspected to have minor bleeding disorders can be circumcised safely in settings where blood products are available.

## Footnotes

* Qualifying notifiable adverse events include complete or partial amputation of the glans or shaft of the penis, tetanus (fatal and nonfatal), any adverse event that results in permanent disability or deformity, any adverse event that results in hospital admission for ≥3 days, or any adverse event that results in death.

## References

[1] Siegfried N, Muller M, Deeks JJ, Volmink J. Male circumcision for prevention of heterosexual acquisition of HIV in men. Cochrane Database Syst Rev 2009;2:CD003362. 19370585 10.1002/14651858.CD003362.pub2 PMC11666075

[2] US President’s Emergency Plan for AIDS Relief (PEPFAR). 2017 PEPFAR latest global results. Fact sheet. Washington, DC: US President’s Emergency Plan for AIDS Relief; 2017. https://www.pepfar.gov/documents/organization/276321.pdf

[3] Bochner AF, Feldacker C, Makunike B, Adverse event profile of a mature voluntary medical male circumcision programme performing PrePex and surgical procedures in Zimbabwe. J Int AIDS Soc 2017;19:21394. 10.7448/IAS.20.1.21394 28362066 PMC5467584

[4] CDC. Voluntary medical male circumcision—southern and eastern Africa, 2010–2012. MMWR Morb Mortal Wkly Rep 2013;62:953–7. 24280914 PMC4585634

[5] Population Services International; College of Surgeons of East, Central and Southern Africa; CDC. Adverse event action guide for voluntary medical male circumcision (VMMC) by surgery or device: 2nd edition. Washington, DC: Population Services International; Arusha, Tanzania: College of Surgeons of East, Central and Southern Africa; Atlanta, GA: US Department of Health and Human Services, CDC; 2017. https://www.malecircumcision.org/resource/adverse-event-action-guide-voluntary-medical-male-circumcision-surgery-or-device-2nd

[6] Project IQ/Jhpiego. Provider verbal pre-screening questions for voluntary medical male circumcision. Baltimore, MD: Project IQ/Jhpiego; 2016. https://www.malecircumcision.org/resource/provider-verbal-pre-screening-questions-voluntary-medical-male-circumcision

[7] National Hemophilia Foundation. Types of bleeding disorders. New York, NY: National Hemophilia Foundation; 2017. https://www.hemophilia.org/Bleeding-Disorders/Types-of-Bleeding-Disorders

[8] World Federation of Hemophilia. World Federation of Hemophilia report on the annual global survey 2015. Montreal, Canada: World Federation of Hemophilia; 2016. http://www1.wfh.org/publication/files/pdf-1669.pdf

